# Acknowledgement to Reviewers of *Brain Sciences* in 2018

**DOI:** 10.3390/brainsci9010007

**Published:** 2019-01-08

**Authors:** Brain Sciences Editorial Office

**Affiliations:** MDPI, St. Alban-Anlage 66, 4052 Basel, Switzerland; brainsci@mdpi.com

Rigorous peer-review is the corner-stone of high-quality academic publishing. The editorial team greatly appreciates the reviewers who contributed their knowledge and expertise to the journal’s editorial process over the past 12 months. In 2018, a total of 214 papers were published in the journal, with a median time to first decision of 17 days and a median time to publication of 40 days. The editors would like to express their sincere gratitude to the following reviewers for their cooperation and dedication in 2018:
Albert, KimberlyMacKenzie, AlasdairAlbonico, AndreaMagnotti, JohnAlexandra, CauquilMaidhof, ClemensAltschuler, RichardMains, Richard E.Ambigapathy, GaneshMajić, TomislavAnandhan, AnnaduraiMakaryus, RanyAnderson, JohnMakin, StephenAndjelkovic, Anuska V.Malmierca, Manuel S.Angelika, SchlarbMännel, ClaudiaAnnen, JitkaMantovani, AntonioApostolidis, ApostolosMantua, JannaAricò, PietroMarchesini, GiulioArinuma, YoshiyukiMariotti, CaterinaArroyave, Nora VanegasMarkofski, WesAust, SabineMårtensson, JohanAzarnia Tehran, DomenicoMartin, Donel M.Baburamani, Ana A.Martinuzzi, AndreaBaldermann, Juan CarlosMasui, KentaBandres-Ciga, SaraMazzone, PaoloBanerjee, AnweshaMcCary, Lindsay M.Barrett, DougMcClung, Colleen A.Basavarajappa, Balapal S.McGarry, AndrewBasirat, AnahitaMehla, JogenderBatarseh, YazanMeloni, Bruno P.Battistella, GiovanniMerikangas, AlisonBeetz, ChristianMessina, AuroraBelfi, AmyMignon, SylviaBerlucchi, GiovanniMiller, Bryan LeeBernardi, GiulioMin, Hoon-KiBeversdorf, DavidMinzenberg, MichaelBianchi, VittorioMiyake, SachikoBilliard, MichelModirrousta, MandanaBishop, LauraMoezzi, BaharBland, Sondra T.Mohapatra, LeenaBloch, YuvalMonson, Brian B.Bogaerts, LouisaMonteiro, SusanaBorgatti, RenatoMontgomery, Erwin B.Botturi, AndreaMontgomery, LaTriceBowling, HeatherMoon, ILSOOBradley, Evan D.Moores, ElisabethBrady-Kalnay, Susann M.Mora, JoanBraun, Ralf J.Moretti, RitaBright, StephenMorrison, MelanieBroccard, Frédéric D.Morse, Anne M.Brondolo, ElizabethMüller, NotgerBrooke, JoanneMunshi, KaizadBrown, Amanda M.Murphy, RonanBruno, JenniferNakanishi, MasakiBrysbaert, MarcNalvarte, IvanBuckley, SueNamgung, UkBukowski, HenrykNavarro Acebes, XavierBurghardt, NeshaNeblett, EnriqueBurke, MelanieNeef, NicoleBuss, Aaron T.Nelson, DavidButler-Barnes, Sheretta T.Neumann, Wolf-JulianCai, ChunyuNewman, LouiseCalcus, AxelleNi, WeimingCampisi, AgataNicoletti, FerdinandoCantello, RobertoNikodemova, MariaCapasso, RaffaeleNolin, SallyCarotenuto, MarcoÖhrfelt, AnnikaCarrus, ElisaOlesnicky, Eugenia C.Casartelli, LucaOosterloo, MaykeCastrén, Maija L.Osier, NicoleCaudle, MichaelPaap, Kenneth R.Cavoretto, PaoloPagonopoulou, OlgaChandaka, Giri KumarPalm, UlrichChandler, Melanie J.Palomba, LetiziaCHERON, GuyPapadelis, ChristosChester, DavidParazzini, MartaCichero, ElenaPark, RebeccaCinel, CaterinaParrott, AndrewCoenen, Volker ArndParthasarathy, SrinivasConstable, PaulPati, DipanwitaCooper-Knock, JohnathanPaul, FriedemannCooperstein, RobertPazzaglia, MariellaCortés Castell, ErnestoPerani, DanielaCox, Christopher R.Peterson, Brian L.Critchley, Hugo D.Pistoia, FrancescaCrowell, AndreaPitt, AnnaCummins, DenisePlatt, Donna M.Curry, TommyPoel, MannesDaikoku, TatsuyaPoulin-Charronnat, BenedicteDaini, RobertaPower, KevinDal Ben, MatteoPulsifer, MargaretDalmaijer, Edwin S.Quarrell, OliverDaverey, AmitaRahimi, FaridDe Berardis, DomenicoRahmathulla, GazanfarDe Deurwaerdère, PhilippeRand, Troy J.De Luca, Maria AntoniettaRaspa, MelissaDe Ruiter, Godard C. W.Redifer, JenniDeeb, WissamRegal, JeanDemetriou, AndreasReichert, ChristophDeriu, FrancaReilly, KarenDesai, AbhishekReimann, Regina R.Dhanasekaran, MuraliRen, QingzhongDidonna, AlessandroReznik, Jacqueline E.Disdier, ClemenceRiccio, PaoloDones, IvanoRocha Ferreira, EridanDowling, W. JayRodolfo, CarloDrigas, Athanasios S.Rodriguez, Francisca S.Dudok, JeroenRodriguez-Contreras, AdrianDufek, Janet S.Rodríguez-Pallares, JannetteD'Urso, GiordanoRodriguez-Revenga, LaiaEdamakanti, ChandrakanthRolston, JohnEggleston, Jeffrey D.Rossignol, JulienEhrich, MarionRoubertoux, PierreEltze, ChristinRoy Choudhury, GouravEnglish, Arthur W.Ruda-Kucerova, JanaEsparham, AnnaRuiz-Gómez, Saúl J.Ethofer, ThomasRusiecki, Jennifer A.Fairclough, StephenSalomone, AlbertoFidler, DeborahSamantaray, SupritiField, MichaelSamsam, MohtashemFigee, MartijnSattler, SebastianFigueras, AlbertSavaskan, Nicolai E.Figueroa-Romero, ClaudiaSchellenberg, E. GlennFirestein, BonnieSchindler, Charles W.Fischer, ItzhakSchwartz, KennethFitzgerald, TerenceScorziello, AntonellaFraga González, GorkaSeeman, Mary V.Franceschini, SandroSellers, KristinFrase, LukasSerafini, GianlucaGe, TianSerniclaes, WillyGershon, TimothySerra, MonicaGervais, NicoleServello, DomenicoGhosh, JayashriSharman, RachelGiammalva, Giuseppe RobertoShaw, Ryan J.Giannakeas, NikolaosSheerin, Christina M.Gilbert, FrédéricShelley-Tremblay, JohnGilger, JeffreyShyman, EricGinsberg, Stephen D.Sidhu, SimranjitGlenn, SheilaSilverdale, MontyGobert, DeniseSimos, PanagiotisGodfrey, Donald A.Sinclair, DuncanGoizet, CyrilSivaraju, AdithyaGoldman-Mellor, SidraSmart, RosannaGoodman, Alan G.Smith, Carolyn BeebeGorgey, Ashraf S.Smith, DeanGorgoni, MaurizioSoucy, Jean-PaulGrahame, Nicholas J.Sparks, Kally O'ReillyGrahn, PeterSt Hilaire, MelissaGreene, Paul E.Stefani, AlessandroGreenfield, JohnStein, JohnGroen, Margriet A.Steinmetz, Peter N.Gross, ChristinaStephens, David N.Grundy, LukeStins, John F.Gu, FengStocker, RetoGu, YianStrano-Rossi, SabinaGuediche, SaraSulek, AnnaGuirguis, AmiraSun, DandanGurrera, Ronald J.Suñer, Damian HeineGurvich, CarolineSurguchov, AndreiGyant, LaVerneSuzuki, JojiHaastert-Talini, KirstenTabernero-Duque, María-JesúsHaavik, HeidiTabolacci, ElisabettaHamano, TadanoriTaghavi, NazitaHanna, AmgadTakaki, ManabuHarper, DanielTakano, KatsuraHauser, Sheketha R.Tanaka, NaoroHeilbronner, UrsTarkar, AartiHellewell, SarahTatsumi, KoukoHelmy, AdelTchanturia, KateHentall, IanTéllez-Zenteno, José F.Hidalgo, JuanTeo, Wei PengHill, Aron TTettenborn, BarbaraHirsch, OliverThomas, CourtneyHoeller, YvonneThompson, BillHordacre, BrentonThornton, RosalindHorn, SebastianToomey, RosemaryHowell, BryanToribio-Mateas, MiguelHu, XiaosuTorres, ClaudioHuang, LeiTronnier, Volker MartinHunt, PamelaTseng, PhillipIacono, DiegoTsoli, MariaIannilli, EmiliaTurban, Jack L.Ibernon, LaureTurnbull, DavidIramina, KeijiTurner, ReneeIrwin, JuliaTuttolomondo, AntoninoJackson, Stephen R.Tyynismaa, HennaJeong, Keun-YeongUnderwood, JonathanJiang, JianxiongUnderwood, Mark D.Jimenez-Shahed, JoohiUsdin, KarenJones, BethVachon, HugoJonsson, Kenisha RussellVale, Fernando L.Junge, CarolineValentín, AntonioKalashnikova, Marinavan der Wel, Robrecht P. R. D.Kamath, VidyulataVeldsman, MicheleKang, ChenVelpula, KiranKarumanchi, AnanthVender, MariaKatsavelis, DimitriosVerhagen Metman, LeoKaul, MarcusVesper, JanKelly, KimberlyViggiano, DavideKhokhar, BilalVladimirov, VladimirKhoutorsky, ArkadyVolk, Alexander E.Kim, Do-YoungVulchanova, Mila DimitrovaKim, Yun-baeW. Stottmann, RolfKitner-Triolo, MelissaWada, NaokiKjellgren, AnetteWalker, Chandler L.Kober, SilviaWalker, Harrison C.Koelman, Johannes H. T. M.Waller, RachelKoldamova, RadosvetaWang, Huei-ShyongKomori, TeruhisaWang, XiaowanKraner, James C.Ward, ChristopherKrause, André FrankWarren, Steven F.Kroll, Judith F.Weaver, Frances M.Kukke, Sahana N.Wellington, CherylKumar, SuneelWelshhans, KristyKupsch, AndreasWestmark, Cara J.Kweon, Oh-KyeongWidhalm, GeorgKwon, Churl-SuWillison, HughLa Vecchia, CarloWinfree, Christopher J.Labuschagne, IzelleWinner, BeateLambert, Sharon F.Wise-Faberowski, LisaLanderos-Weisenberger, AngieWiseheart, RebeccaLangevin, Jean-PhilippeWolter, TilmanLangford, T. DianneWood, Matthew D.Levy, YonataWooden, JessicaLewis, GwynXiao, LiLi, LinXiao, RongLi, Shengwen CalvinXiong, YeLi, YinlinXu, HuaxiLiapi, CharisYamazaki, YuLindstrom, Jon MartinYang, YuanLombardi, AngelaYoong, MichaelLong, Nicole M.Young, KayleneLopez Lopez, DanielYoung, KymberlyLoui, PsycheYoussofzadeh, VahabLovrecic, MercedesYstrom, EivindLu, Chia-FengZadeh, GelarehLubetzky, Anat V.Zaehle, TinoLui, FaustaZahr, Natalie M.Lupo, GiuseppeZhou, MuMabbott, Neil


